# Development of efficient targeted insertion mediated by CRISPR-Cas12a and homology-directed repair in maize

**DOI:** 10.3389/fgeed.2025.1713347

**Published:** 2025-12-04

**Authors:** Brenden Barco, Shujie Dong, Yuki Matsuba, Ashley Crook, Ruiji Xu, Yingxiao Zhang, Chengjin Zhang, Ryan Carlin, Kevin Potter, Stephen B. Rigoulot, Jeongmoo Park, Erin M. Seaberry, Allison Parrish, Sivamani Elumalai, Sam Nalapalli, Craig Schuller, Anna Prairie, Anna Mangum, Kangfeng Mei, Hao Wu, Melissa Murray, Kristin Setliff, Francine Johnson, Dawn McNamara, Ling Zhu, Mark Rose, Weining Gu, Hao Hu, Yuanji Zhang, Yaping Jiang, Wenling Wang, Guozhu Tang, Lizhao Geng, Jianping Xu, Wan Shi, Jason Nichols, Tim Kelliher, Liang Shi, Ian Jepson, Qiudeng Que

**Affiliations:** 1 Syngenta Crop Protection, LLC, Research Triangle Park, Schaumburg, NC, United States; 2 Primary Products Ingredients Americas LLC (Primient), Schaumburg, IL, United States; 3 Meiwenti AgBio Ops and IP Solutions, Cary, NC, United States; 4 Retired, Raleigh, NC, United States; 5 Retired, Cary, NC, United States; 6 Syngenta Biotechnology China Co., Ltd., ZhongGuanCun Life Science Park, Changping, Beijing, China; 7 North Carolina State University, Raleigh, NC, United States; 8 Retired, Chapel Hill, NC, United States

**Keywords:** targeted insertion, maize, CRISPR-Cas12a, homology-directed repair, gRNA screening, nanopore sequencing

## Abstract

Targeted insertion (TIN) of transgenic trait cassettes has the potential to reduce timeline and cost for GM product development and commercialization. Precise genome engineering has made remarkable progress over the last several decades, particularly with the use of site-directed nucleases as core editing machinery. However, there are still many critical factors that can impact TIN efficiency including insertion site selection, nuclease optimization and expression, donor vector design, gene delivery, and stable event regeneration. Here, we develop workflows for target site sequence identification and gRNA screening for CRISPR-Cas12a system and demonstrate its successful application for TIN in maize with donor sequences up to 10 kilobase pairs (kb) in size. We first prioritize genomic regions for inserting transgenes *in silico* using bioinformatics tools and then test gRNA performance using a leaf protoplast transient assay. Despite its known low efficiency, we identify homology-directed repair (HDR) as the preferential pathway for directing targeted insertions of large sequences in immature embryos and demonstrate double-junction integrations at a rate of up to 4%. We further apply a molecular analysis workflow using large amplicon TaqMan assays and nanopore sequencing for streamlined identification and characterization of high-quality insertion events with intact large inserts. Analysis of TIN events across generations suggests that efficiency bottlenecks are not limited to donor targeted integration; attrition in efficiency also results from partial or additional donor insertion, chimerism, and close linkage with undesired sequence insertions such as those encoding the editing machinery. This work represents a major step forward in realizing the potential of precise genome engineering in maize for basic research and biotech trait development applications.

## Introduction

Genetically modified (GM) traits in field crops have long been generated by means of random integration into the genome. Transformation through random insertion can be highly efficient in generating single copy intact insertions, but it still has several potential issues including transgene expression variation and endogenous gene disruption. The variability across integration locations, commonly known as position effects, frequently leads to high rates of GM event attrition due to challenges of poor trait expression and/or introgression (Meyer, 1995; Mumm and Walters, 2001). By contrast, targeted insertion (TIN) of large DNA sequences into desirable genomic locations could enable the development of more consistently performing GM traits without disrupting endogenous genes. The potential cost saving from the use of TIN in commercial event characterization, field trialing, and trait stacking makes it an attractive tool for crop improvement.

A common strategy for enabling TIN relies on direct insertion of transgenes into DNA double strand breaks (DSBs) generated by site-directed nucleases (SDNs) via endogenous DNA repair pathways. Several DSB repair mechanisms have been used to enable large sequence insertions in eukaryotes: non-homologous end joining (NHEJ, also known as canonical-NHEJ), microhomology-mediated end joining (MMEJ, or alternative-NHEJ), and homology-directed repair (HDR) (Puchta et al., 1996; Chilton and Que, 2003; Nakade et al., 2014; Dong OX. et al., 2020). NHEJ is the dominant DSB repair pathway in plants and is error-prone, introducing random nucleotide insertions and deletions (InDels) at the repair site (Manova and Gruszka, 2015). By contrast, HDR utilizes a homologous template, e.g., a sister chromatid or exogenously supplied template, to achieve a precise repair of the break (Puchta et al., 1996). MMEJ features a blend of these pathways, leveraging end resection similarly to HDR by way of microhomology, but nonetheless remaining an error-prone process, particularly near the DSB site (Deriano and Roth, 2013). Due to its seamless nature, HDR is favorable for targeted gene replacement or TIN of large DNA sequences in plants. However, HDR’s requirement of *de novo* DNA strand synthesis in most plant tissues is thought to lead to lower effective integration efficiencies relative to end-joining pathways that leverage a “cut-and-paste” approach (Puchta, 2005). Furthermore, HDR and MMEJ are generally restricted to shorter phases of active cell division (G2/S and M respectively), whereas NHEJ is active during the typically longer G1 phase (Beucher et al., 2009; Karanam et al., 2012; Brambati et al., 2023). As a result, the baseline activity of these pathways varies based on the tissue type used for transgene delivery and even across individual cells. The efficiency of all three classes of pathways have not been systematically compared for directing large sequence TINs in a field crop.

Over the last 2 decades, several types of SDNs with customizable recognition specificity including zinc finger nuclease (ZFN), transcription activator-like effector nuclease (TALEN), engineered meganuclease and clustered regularly interspaced short palindromic repeats (CRISPR)-Cas9 endonuclease have become available for use in plant genome editing (Voytas, 2013; Gao et al., 2010). These flexible site-directed nucleases, especially programmable RNA-guided CRISPR-Cas ribonucleoproteins (RNPs) have made it possible to directly insert large transgenes into desirable chromosomal loci despite the relatively low and variable efficiency in crop genomes (Svitashev et al., 2015; Endo et al., 2016; Shi et al., 2017; Danilo et al., 2018; Hummel et al., 2018; Dong et al., 2020; Lu et al., 2020; Dong O.X. et al., 2021; Kumar et al., 2022). Several examples of higher TIN efficiencies mediated by ZFN and Cas9 have been achieved in maize via particle bombardment and *Agrobacterium*-mediated delivery (Shukla et al., 2009; Gao et al., 2020; Barone et al., 2020; Peterson et al., 2021).

Although inroads have been achieved regarding SDN-mediated large sequence TIN in crops, several aspects still require further study and improvements to make the identified factors transferable to other systems. One is the use of donors featuring multiple trait gene cassettes. In such cases, TIN rates are usually much lower in comparison with donors featuring selectable marker cassettes alone. Event quality for multi-cassette TIN is another aspect. Many targeted insertion events generated via HDR do not contain intact, clean, single copy, and seamlessly integrated donor sequence at both insert ends. Instead, a large percentage of TIN events contain truncated partial inserts, or else complex outcomes featuring multiple transgene copies and stuffer DNA fragments resulting from a mixture of HDR and non-homologous end-joining pathways at each DSB side (Puchta, 1998; Peterson et al., 2021). Finally, the effect of variable DNA delivery methods on targeted insertion efficiency and event quality has not been directly compared by any report.

We and others previously reported efficient targeted mutagenesis in maize using Lachnospiraceae bacterium ND2006 Cpf1/Cas12a (LbCpf1/LbCas12a) and *Acidaminococcus sp*. Cpf1/Cas12a (AsCpf1/AsCas12a) delivered by RNP or expressed from a transgene (Lee et al., 2019; Dong S. et al., 2021). Cas12a-mediated gene targeting (allele replacement) and TIN have been reported in rice when the editing machinery was expressed from DNA vectors delivered by particle bombardment (also known as biolistics; Begemann et al., 2017; Li et al., 2018; Li et al., 2020) or expressed from a viral vector in tomato (Vu et al., 2020). However, so far there is no report of Cas12a-mediated targeted insertion in maize, either delivered as RNP or expressed from a transgene. Here, we systematically evaluate and optimize the efficiency of large sequence insertions in maize using Cas12a-mediated genome editing. We investigate several key aspects, including the selection of guide RNAs for editing, and a comparison of different DNA repair pathways, delivery methods, and TIN donor preparations to enhance efficiency. We evaluate the effect of Cas12a high-activity variants, binary vector, and donor DNA configuration on quality event generation efficiencies through two generations. Finally, we characterize insertion events using nanopore long-read sequencing technology to understand the detailed molecular outcomes of the editing and repair process. Our results suggest the bottleneck of achieving high efficiency TIN via HDR extends not just to insertion rates in T0 events but also to the rate of quality inserts. This comprehensive framework for evaluating and optimizing large sequence insertions in maize can be applied to other crop species and contributes to the advancement of crop improvement through precision genome engineering.

## Results and discussion

### Process flow for selection of gRNAs for targeted insertion

CRISPR-Cas12a systems have been shown to mediate efficient genome editing in plants. However, the editing efficiencies showed large nuclease- and target-dependent differences (Bernabé-Orts et al., 2019; Dong S. et al., 2021). To improve the success rate of targeted insertion studies, we aimed to develop a process workflow for selecting high performance gRNAs and editing targets, starting with candidate gRNA selection followed by transient editing assays. We previously demonstrated efficient transient editing near the insertion site of the commercial transgenic event MIR604 (approximate position 38.9 Mb on Chr1, B73 RefGen_v5) which provides corn root worm control (Dong S. et al., 2021). MIR604 insert-neighboring regions (aka ZmSH1 locus) were selected as a testing platform for building the process workflow. We selected a 4,915 base pair (bp) region that overlapped with the insertion site while staying distal to gene models and repeat-heavy regions and identified 117 gRNAs with a TTTV protospacer adjacent motif (PAM) and moderate GC content (30%–80%). We chose a subset of these gRNAs based on predicted gRNA structure and potential off-target sites for further analysis via an *in vivo* transient assay, which consists of high-throughput transfection of RNP complexes to protoplasts derived from etiolated maize leaves (Rigoulot et al., 2023). Transient experiments were performed using gRNAs assembled from LbCas12a-Ultra (see Methods) and editing target analysis was performed by amplicon deep sequencing. The improved gRNA selection and *in vivo* editing activity assay workflow led to the identification of many candidates with editing efficiencies on par with ZmSH1gRNA2 and the benchmark control Bx9TS2 (Supplementary Table 1).

Editing efficiencies ranged considerably across the gRNAs tested in protoplast. To better understand this variability, we evaluated methylation levels at the sites corresponding to the gRNA targets using previously generated whole-genome bisulfite sequencing leaf tissue data in NP2222 genotype. CHG and CpG methylation signals were weakly predictive of editing rate (*R* ^2^ = 0.40 and 0.37, respectively), whereas CHH methylation signal was low overall and not predictive (*R* ^2^ = 0.09). Interestingly, all gRNAs with undetectable editing rates resided in regions of high (>85%) CHG and CpG methylation (Supplementary Table 1). This dataset mirrors similar observations in plants using Cas9 (Weiss et al., 2022) and indicates that genome-wide DNA methylation signals can be used as an additional tool to screen out poor-performing LbCas12a gRNAs.

DNA vectors were built for several gRNAs to validate editing rates in stable maize T0 events. In addition to Cas12a and gRNA expression cassettes, these vectors contained a phosphomannose isomerase (PMI) selectable marker. Four gRNAs with a range of performance were selected for this evaluation and transgenic events were generated through biolistics of isolated maize immature embryos and mannose selection as described (Dong, S et al., 2021). Mutagenesis at the four different target sites was assessed through high-throughput TaqMan assays (Chen et al., 2018; Dong S. et al., 2021). The stable maize editing rates (calculated by the number of edited events divided by total transgenic event number) were broadly reminiscent of observations from the transient assay, with editing rates ranging from 25% to 83.8% (Supplementary Tables 1, 2). It should be noted that in comparison with RNP-mediated transient editing in protoplasts, stable transgenic plants will have a longer time to edit the target sites after the transformation process until the target sequence is fully mutated or else until the inserted Cas12a transgene is removed through breeding. Also, the target sequences’ chromatin structure might be different in immature embryos vs. young leaf protoplast. These differences may explain the discrepancy in transient and stable editing efficiency. The most efficient gRNA (ZmSH1gRNA2) for stable editing was subsequently selected for evaluation of large sequence insertions via targeted insertion experiments in immature embryos.

### Targeted large sequence insertions in maize immature embryos are efficiently integrated via homology directed repair (HDR)

**FIGURE 1:**
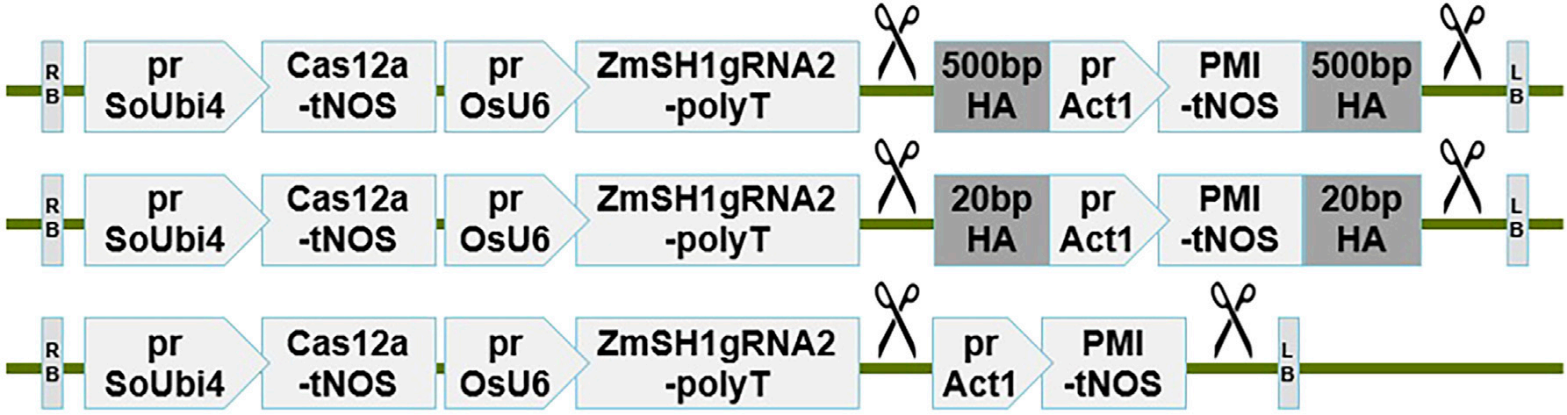
Schematic of all-in-one Cas12a editing machinery and donor constructs for Agrobacterium-mediated maize transformation. HA, homology arm of varying size. Scissors indicate ZmSH1gRNA2 cut sites to enable donor release. prSoUbi4, sugarcane Ubiquitin-4 promoter; Cas12a, maize codon-optimized eMb2Cas12a coding sequence; tNOS, NOS terminator; prOsU6, rice U6 promoter; prAct1, rice actin-1 promoter; PMI, phosphomannose isomerase coding sequence; RB, T-DNA right border; LB, T-DNA left border.

CRISPR-Cas cleavage-mediated gene insertion facilitated by native DNA repair pathways are frequently challenged by low efficiencies. HDR and NHEJ have been employed for CRISPR-Cas-mediated large donor TIN in maize immature embryos (Svitashev et al., 2015; Gao et al., 2020; Barone et al., 2020; Peterson et al., 2021; Anderson, 2023), however, it remains unclear which pathway is the most efficient for this target tissue. Also, the optimal length of homology arms has not been directly tested for targeted insertion in maize. We therefore compared TIN rates leveraging HDR, MMEJ, and NHEJ by designing all-in-one machinery and donor constructs featuring varying lengths of homology arms corresponding to the ZmSH1gRNA2 target site. Efficient targeted integration of the 3.7 kb sequence via HDR, MMEJ, or NHEJ would lead to generation of insertion-positive events using a 500 bp, 20 bp, or 0 bp homology arm design, respectively (Figure 1). Transgenic events were generated through *Agrobacterium*-mediated delivery and mannose selection. To detect insertions at the target site, TaqMan-based assays were run at both 5′ and 3′ junctions (Chen et al., 2018). Events positive for insertions at either junction were further analyzed via gel-based assays (see Methods). While transformation frequency and editing rate remained consistent across the three constructs (15.2%–23.1% and 77.5%–83.5% ranges, respectively), the TIN rate (defined as the number of events positive for HDR at one or both junctions via gel-based polymerase chain reaction [PCR] out of all PMI-positive events) ranged widely (Table 1). Of 957 and 1296 events generated from transformation constructs with the NHEJ (pSYN29514) and MMEJ (pSYN29515) designs respectively, few single TIN-positive events were recovered with overall TIN rates (ajTIN%) of 1.5% and 0.8%, respectively. By contrast, the HDR construct (pSYN29527) yielded an ajTIN rate several fold higher (4.2%). Similar trends were observed for the quality TIN rate (djTIN%) across the constructs. It should be noted that Cas12a-mediated donor release with ZmSH1gRNA2 generates sticky ends of five nucleotides with homology to the chromosomal target. Even though the sticky ends are likely resected by the host exonuclease activity thus reducing the sticky end length, it is also possible that the “0” bp homology donor is inserted via MMEJ-mediated mechanism.

**TABLE 1:** Targeted insertion (TIN) efficiency at chromosomal target ZmSH1gRNA2 with constructs containing different lengths of homology arms.

HOM arm length (bp)	Insert size (bp)	Construct	Experiments	Explants	PMI-positive events (TF%)	Edited events (rate%)	Any junction PCR-positive TIN events (ajTIN rate%)	Double junction PCR-positive TIN events (djTIN rate%)
526/507	3674	pSYN29527	12	9501	1949 (20.5%)	1576 (80.9%)	81 (4.2%)	39 (2.0%)
20/20	3674	pSYN29515	6	8512	1296 (15.2%)	1004 (77.5%)	20 (1.5%)	3 (0.2%)
0/0	3674	pSYN29514	5	4137	957 (23.1%)	799 (83.5%)	8 (0.8%)	1 (0.1%)

All constructs express eMb2Cas12a editing machinery and events were generated using Agrobacterium-mediated transformation. Transformation frequency (TF%) = (Number of PMI-positive events/Number of starting explants) × 100%; Editing rate (%) = (Number of edited events / Number of PMI-positive transformants) × 100%; Any-junction targeted insertion rate (ajTIN%) = (Number of PCR, positive events at either left or right junction/ Number of any copy events) × 100%; Double-junction targeted insertion rate (djTIN%) = (Number of PCR, positive events at both left and right junctions/ Number of any copy events) × 100%.

In addition to *Agrobacterium*-mediated delivery, we also tested biolistic delivery for TIN using donor fragments with 50 bp and ∼500 bp length homology arms with co-delivered with a vector expressing LbCas12a and ZmSH1gRNA2 (Supplementary Table 3). While transformation and editing frequencies of donor fragments with 50 bp and 500 bp homology arms were in a roughly similar range, the TIN rates increased with larger homology arm length (Supplementary Table 3). In summary, results with both biolistic and *Agrobacterium*-mediated transformation methods and two different Cas12a systems indicate that HDR is the preferred pathway over NHEJ and MMEJ for directing large intact donor sequence insertions to Cas12a nuclease cleavage sites in immature maize embryo explants.

### Donor size impacts HDR-mediated targeted insertion efficiencies

Delivery of large (greater than 10 kb) sequences comprising multi-trait molecular stacks is highly desirable for GM product development. While HDR efficiency has been observed to be negatively associated with insert size in mammalian cells (Li et al., 2014), similar systematic studies have not been carried out in plants (Chen et al., 2022). We therefore evaluated the effect of various sized HDR payloads on transgene insertion rates by co-delivering RNP and donor sequences with lengths of 6,177 bp, 7,508 bp and 10,182 bp, excluding homology arms (Supplementary Table 4). Transformation frequency was impacted by the variation in overall construct size, in agreement with previous findings (Frary and Hamilton, 2001). From a minimum starting point of approximately 4000 immature embryo explants per construct, the rate of targeted insertion decreased with increasing HDR donor size, achieving rates of 0.9%, 0.5% and 0.3% for the three respective sizes. These results indicate that increased donor size as delivered through biolistics is associated with a decreased TIN rate and transformation frequency.

### Improvement of HDR-mediated cassette insertion with biolistic delivery

One potential limiting factor for efficient HDR is the available amount of homologous donor template molecules. To test the impact of donor concentration on targeted insertion rates, we designed experiments using biolistic co-bombardment of DNA and RNP as a delivery method wherein gel-purified linear donor DNA restriction fragments were introduced. We observed that up to 0.76 pmol of donor molecules could be effectively delivered to each Petri plate of immature embryo explant targets with no reduction in transformation frequency or editing rate (Supplementary Table 5). Moreover, we observed a 3.3-fold increase in TIN rates for treatment with 0.76 pmol donor molecules compared to treatment with 0.38 pmol donor molecules, indicating that HDR-based insertion is responsive to the amount of donor template introduced by biolistics.

Phosphorothioate modifications to the deoxyribose-phosphate backbone can be employed to counter degradation of DNA donors by endogenous nucleases. Incorporating these modifications to small numbers of terminal nucleotides leads to improvements in targeted insertion frequencies, however these applications are usually limited to oligonucleotides of 200 or fewer nucleotides in length (Renaud et al., 2016). A recent report demonstrated successful targeted insertion of promoter sequences up to 2 kb, wherein 5′end modifications were incorporated via PCR with chemically modified primers (Lu et al., 2020). However, the degree to which chemical modifications improve insertions of even larger sequences was not explored in that study, and the effects of end modification on insertion of whole gene cassettes were unknown.

To test the hypothesis that DNA donor stability plays an important role for larger sized donor insertions, we generated PCR amplicons of a PMI marker gene expression cassette plus left and right homology arms (4.7 kb total size) corresponding to another selected genomic region (see Methods). Primers featured two phosphorothioate linkages at the 5′-most end with the remaining sequences having regular 5′phosphate linkages. As a control, we generated PCR amplicons of the PMI cassette lacking these modifications. Supplementary Table 6 shows the stable transformation, editing and TIN results of modified and unmodified DNA donor sequences delivered by biolistics. The transformation frequency for both donors fell within a similar range, as did the editing efficiencies. By contrast, the djTIN rate increased 5-fold with with the end-protected donor fragment (0.2% for unmodified compared to 1.0% for modified). Collectively, these data indicate that modifications to donor chemistry and concentration can increase quality TIN rate (djTIN%) via HDR using biolistic delivery.

### Evaluation of targeted insertion efficiencies by genetic transformation method

*Agrobacterium* and biolistics are the two most widely used methods of plant genetic transformation (Vain, 2007; Que et al., 2019). To better understand which approach is more efficient for HDR-mediated targeted insertion, we compared these delivery methods across five delivery conditions using an identical donor PMI expression cassette (Table 2). Two *Agrobacterium* strains containing the supervirulent helper pVGW7 (Zhong et al., 2018) were evaluated–the commonly used LBA4404 strain as well as Chry5d2, a fully disarmed version of Chry5 strain (Chen et al., 2019). Three particle bombardment experimental conditions were tested. One experiment delivered DNA constructs expressing the CRISPR-LbCas12a system containing a temperature-tolerant D156R mutation (LbCas12a-D156R, Kleinstiver et al., 2019; Schindele and Puchta, 2019). Two other experiments used different Cas12a RNPs, one with AsCas12a-Ultra and another with LbCas12a-Ultra (see Methods). Results of these stable transformation experiments are shown in Table 2. Of the two versions of RNP delivered, AsCas12a-Ultra yielded notably lower editing rates and no TIN events were recovered.

**TABLE 2:** A comparison of delivery methods at ZmSH1gRNA2.

Delivery method	Enzyme	Experiments	Explants	PMI-positive events (TF%)	Quality events (SCBBF rate%)	Edited events (rate%)	Any junction PCR-positive TIN events (ajTIN rate%)	Double junction PCR-positive TIN events (djTIN rate%)
Agro: Chry5d2	LbCas12a D156R	17	8599	1036 (12.1%)	257 (46.2%)	504 (48.6%)	25 (2.4%)	21 (2.0%)
Agro: LBA4404	LbCas12a D156R	6	6136	584 (9.5%)	76 (48.4%)	276 (47.3%)	14 (2.4%)	9 (1.5%)
RNP biolistics	AsCas12a Ultra	3	2451	156 (6.4%)	n.d	43 (27.5%)	1 (0.6%)	0 (0%)
RNP biolistics	LbCas12a Ultra	3	2300	214 (9.3%)	n.d	90 (42.0%)	8 (3.7%)	3 (1.4%)
DNA biolistics	LbCas12a D156R	15	12567	1323 (10.5%)	72 (14.1%)	960 (72.5%)	102 (7.7%)	53 (4.0%)

All experiments utilize construct pSYN27413, featuring a 2-T-DNA, 2-cut design. Transformation frequency (TF%) = (Number of PMI-positive events/Number of starting explants) x 100%; Editing rate (%) = (Number of edited events/ Number of PMI-positive transformants) x 100%; SCBBF, rate (%) = (Number of events with a single copy of the selectable marker and vector backbone-free [SCBBF]/ Number of any copy events sampled for backbone); Any-junction targeted insertion rate (ajTIN%) = (Number of PCR, positive events at either left or right junction/ Number of any copy events) x 100%; Double-junction targeted insertion rate (djTIN%) = (Number of both junction PCR, positive events at both left and right junctions/ Number of any copy events) x 100%.

The *Agrobacterium* treatments yielded TF, editing, and djTIN rates slightly higher than LbCas12a-Ultra RNP delivery. Agrobacterium treatments in general also resulted in similar rates of djTIN and total TIN, whereas biolistic treatments exhibited a lower djTIN rate relative to total TIN (Table 2). This phenomenon can be explained by previous observations that a higher degree of fragmented transgenes results from biolistic delivery (Chilton, 1993), which leads to a higher rate of low-quality events.

The Chry5 *Agrobacterium* strain is known for its high virulence and is associated with high transformation efficiencies in dicots (Grant et al., 2003; Ko et al., 2004) and monocots (Chen et al., 2019). Our experiments using Chry5d2 strain relative to the more commonly used LBA4404 confirmed these findings, as we observed a slightly higher TF and slightly lower multi-copy event rate and backbone-free rate (Table 2). Moreover, the editing rate and djTIN% were also higher in this strain compared to LBA4404 (Table 2). These results suggest that Chry5d2 is a preferred strain for Cas12a-mediated targeted insertion due to its high virulence, in agreement with previous results using TALENs (Chen et al., 2019).

In contrast to RNP- or *Agrobacterium*-mediated delivery, DNA biolistics yielded the highest djTIN and total TIN rates of all approaches (4.0% and 7.7%, respectively). Three factors can explain this efficiency gap. Relative to *Agrobacterium*-mediated delivery which delivers at most ∼10 copies of a transgene of interest (Dickinson et al., 2023), biolistics introduces several orders of magnitude more copies of template, and as shown in Supplementary Table 5, our observed efficiency of HDR-mediated integration in maize scales upwards with the availability of donor template. However, this observation does not explain why RNP and DNA-mediated deliveries - which utilize the same amount of donor DNA–yield such large observed differences in djTIN efficiencies. Indeed, delivery of a preformed CRISPR RNP would be expected to outperform CRISPR DNA delivery in terms of TIN% since the former molecules can more rapidly initiate DSBs when large number of donor molecules are also present in the nucleus, a critical factor for successful HDR. A second factor which explains the observed efficiency gap across biolistic delivery methods relates to the process of complexing and precipitating mixtures of genome editing reagents onto the gold particles. Precipitation of DNA onto gold particles is a well-established method for biolistic delivery. By contrast, the method for RNP assembly and loading onto gold particles is less optimized, less efficient and more variable. For example, the process of dehydrating RNP at ambient temperatures might lead to a high rate of degradation or misfolding. Finally, differences in the LbCas12a protein sequence used in RNA and DNA delivery may provide a further explanation for the gap between DNA and RNP delivery rates.

Compared to *Agrobacterium* delivery which utilizes easily scalable liquid cultures, biolistic delivery often requires individual bombardments of each sample which greatly limits its throughput. Therefore, despite the higher rates of insertion observed with DNA-based biolistics, we opted to use *Agrobacterium-*mediated gene delivery utilizing the Chry5d2 strain in subsequent experiments due to its simplicity and ease of operation.

### Improved Cas12a activity and binary vector design further enhance insertion efficiency

Integration efficiency mediated by HDR depends in part on the timing of DSB initiations during and after gene delivery. A well-expressed and highly active site-directed nuclease is therefore critical to enable effective large sequence insertions. As a result, we sought to further enhance editing efficiencies by leveraging a Cas12a ortholog from *Moraxella bovoculi* AAX08 (Mb2) optimized for genome editing in plants. The Mb2Cas12a ortholog was previously demonstrated to edit to high frequency at low temperatures in rice (Zhang et al., 2021); However, its activity in maize was low (results not shown). We improved WT Mb2Cas12a′s editing activity in maize by inserting a long linker design next to the NLS at the N-terminus, a short linker with 2 NLS at the C-terminus and an intron in the coding region (Table 3). In addition, rational design-based engineering was performed in several domains to improve the activity of Mb2Cas12a (Geng et al., 2024). We evaluated two such engineered Mb2Cas12a variants (eMb2Cas12a-Opt1 and -Opt2, encoded on pSYN28303 and pSYN28304) alongside the wild-type enzyme (encoded on pSYN27848) for targeted insertion at ZmSH1gRNA2 (Table 3). The transformation frequency for constructs featuring eMb2Cas12a-Opt1 and -Opt2 (11.5% and 13.6% respectively) was lower than the construct featuring the wild-type enzyme (21.8%), suggesting possible mild toxicity. Aside from this difference in transformation frequency, we did not observe any obvious phenotypic effects. All Mb2Cas12a vectors achieved editing rates in a roughly similar range, although the fraction of events with biallelic edits was slightly higher for the eMb2Cas12a-Opt1 and -Opt2 (69.5% and 68.6% respectively) compared to wild-type Mb2Cas12a with a long linker and intron (61.6%). Additionally, while we failed to obtain any djTIN-positive event when the wild-type Mb2Cas12a was used, many such events were generated from the constructs expressing eMb2Cas12a-Opt1 and -Opt2 (Table 3).

**TABLE 3:** Effect of eMb2Cas12a amino acid mutations and T-DNA designs on editing and targeted insertion rates.

Construct	Cas12a ortholog & point mutations	BH & Nuc domains	Design	Experiments	Explants	PMI-positive events (TF%)	Edited events (rate%)	Biallelic edited events (rate%)	Double junction PCR-positive TIN events (djTIN rate%)
pSYN27848*	Mb2Cas12a, WT	WT	1T2C	4	989	216 (21.8%)	166 (76.9%)	133 (61.6%)	0 (0%)
pSYN28303	eMb2Cas12a “Opt1”, E797A, F357W, V921K	From AsCas12a	1T2C	7	3002	344 (11.5%)	275 (79.9%)	239 (69.5%)	7 (2.0%)
pSYN28304	eMb2Cas12a “Opt2”, E797A, F357W	From AsCas12a	1T2C	9	4664	636 (13.6%)	492 (77.4%)	436 (68.6%)	15 (2.4%)
pSYN28315	eMb2Cas12a “Opt1”, E797A, F357W, V921K	From AsCas12a	2T2C	11	5544	636 (11.5%)	338 (53.1%)	293 (46.1%)	18 (2.8%)
pSYN28316	eMb2Cas12a “Opt2”, E797A, F357W	From AsCas12a	2T2C	8	4391	580 (13.2%)	280 (48.3%)	250 (43.1%)	8 (1.4%)
pSYN27413*	LbCas12a, D156R	WT	2T2C	5	1962	331 (16.9%)	188 (56.8%)	140 (42.3%)	5 (1.5%)

1T2C, binary vector design with only 1 T-DNA, for delivering both Cas12a editing machinery expression cassettes and donor, but using 2 Cas12a-mediated cuts to release donor sequences (as shown in Figure 1); 2T2C, binary vector design with 2 T-DNAs, one T-DNA, for delivering Cas12a editing machinery expression cassettes and another T-DNA, for delivering donor sequence which is later released from the second T-DNA, by two cuts of Cas12a-mediated cleavage. Asterisks denote presence of the potato ST-LS1, intron in the Mb2Cas12a CDS (pSYN27848) and the first intron of Arabidopsis *CHC1* gene (AT5G14170) in the LbCas12a CDS (pSYN27413).

Addition of the Cas12a and gRNA cassettes greatly increases the size of binary vector T-DNAs, a factor known to negatively impact intact T-DNA delivery and transformation efficiency (Park et al., 2000). We subsequently hypothesized that placement of editing machinery and HDR donor on separate and shorter T-DNAs could lead to improved overall transgene delivery. To investigate this possibility, we tested the impact on TIN of transformation with a two T-DNA vector design (Supplementary Figure 1). This design splits the single large T-DNA (14.7 kb) into two smaller ones: the first T-DNA carries the CRISPR-Cas12a machinery (7.1 kb) and the second T-DNA carries the PMI selectable marker and a gene of interest (8.0 kb). Three versions of editing machineries, eMb2Cas12a-Opt1 and -Opt2, and LbCas12a-D156R mutants were tested using these designs. We did not observe any changes to transformation frequency from a two T-DNA design, and editing rates dropped by almost half, suggesting a sizable portion of events did not receive the machinery T-DNA (Table 3). To explore this possibility further, we calculated a separate editing rate comprising the number of editing-positive events out of events positive for both the selectable marker and Cas12a machinery. By this metric, editing across both one and two T-DNA constructs fell within a higher and more narrow range, confirming that the observed differences in editing rate are attributable to marker placement in the different T-DNA designs (Supplementary Table 7). Comparison across Cas12a versions revealed that their editing rates were similar as well, although the eMb2Cas12a-Opt1 variant consistently yielded the highest editing rate (Supplementary Table 7). Additionally, this mutant yielded the highest biallelic editing rate among the two T-DNA constructs tested, as well as the highest double junction positive event rate (djTIN rate%, Table 3). For these reasons, we took eMb2Cas12a-Opt1-associated constructs forward for further analysis.

### Binary vector design with 2 T-DNAs improves rates of clean targeted insertion events

A second rationale for employing multiple T-DNAs on one binary vector is the ability to generate “clean” events (i.e., free of undesirable sequences, for instance selectable markers or genome editing machinery, Komari et al., 1996). We therefore evaluated the rate by which clean pSYN28303 and pSYN28315 events could be recovered by backcrossing mature T0 events and analyzing progeny (BC1). We defined a clean event as carrying no un-segregable insertion of CRISPR-Cas12a machinery, randomly integrated donor copies, and other undesirable sequences such as vector backbones, based on TaqMan assay analysis.

Of the 7 and 18 djTIN-positive T0 events generated from pSYN28303 and pSYN28315 transformations respectively (Table 3), all but two events from pSYN28315 survived in the greenhouse and produced viable seed, thus achieving seed-set rates of 100% and 88.9% respectively (Table 4; Supplementary Table 8). From these materials, up to 32 BC1 plants per T0 event were analyzed. Of the events that produced BC1 seeds, six from pSYN28303 events and 13 from pSYN28315 contained at least 1 BC1 segregant positive for the targeted insertion at both junctions, netting heritability rates of 85.7% and 81.2% respectively (Table 4; Supplementary Table 8). Of these, two pSYN28303 events and eight pSYN28315 events produced at least one clean BC1 segregant (Supplementary Table 8). The one T-DNA design thereby achieved a BC1 clean insertion rate of 33.3% compared to 61.5% for the two T-DNA design (Table 4). Progeny associated with clean events exhibited Mendelian inheritance patterns at the targeted insertion locus (1:1 ratio expected from backcrossed hemizygous insertions, chi-square p-values >0.05, Supplementary Table 9).

**TABLE 4:** End-to-end TIN efficiency based on five attrition metrics: transformation frequency, targeted insertion rate, seed set rate, BC1 heritability rate, and BC1 clean insertion rate.

Construct ID (binary vector design)	pSYN28303 (1T2C)	pSYN28315 (2T2C)
a. Number of starting immature embryo explants used	3002	5544
b. Number of T0 (PMI+) transgenic events	344	636
c. Transformation frequency, [(b/a)] × 100%	11.5%	11.5%
d. Number of all djTIN events	7	18
e. djTIN efficiency (T0), [(d/a)] × 100%	0.2%	0.3%
f. djTIN rate (T0), [(d/b)] × 100%	2.0%	2.8%
g. Clean djTIN events	3	5
h. Clean T0 djTIN rate, [(g/d)] × 100%	42.9%	27.8%
i. Number of events analyzed for heritability	7	16
j. Seed set rate	100.0%	88.9%
k. BC1 djTIN	6	13
l. djTIN heritability rate (BC1), [(k/i)] × 100%	85.7%	81.3%
m. Clean djTIN events (BC1)	2	8
n. Clean BC1 rate, [(m/k)] × 100%	33.3%	61.5%
o. Clean heritable djTIN rate [(m/b)] × 100%	0.58%	1.25%
p. E2E clean djTIN efficiency, [(m/a)] × 100%	0.07%	0.14%

Using the metrics described in Table 4, an operational end-to-end efficiency was calculated, consisting of the number of events with one or more clean BC1 progenies out of all starting T0 explants. We used this metric to compare the impact of the one vs. two T-DNA designs. While the end-to-end clean TIN efficiencies were low (well under <1%) in either case, the two T-DNA design featuring separate HDR donors and machinery was twice as efficient at generating clean and heritable insertions in the next-generation (Table 4).

Since both one and two T-DNA designs feature donors flanked by gRNA cut sites for release and targeted integration, it is interesting to speculate as to why two T-DNAs further enhance the clean integration rate. Given the selectable marker’s presence in the donor template of the two T-DNA design, transient editing and integration might occur early following gene delivery such that machinery-free TIN-positive events could be recovered. However, we did not observe higher overall clean T0 rates using two T-DNAs (Table 4). A more likely explanation is that the lesion following donor release in a one T-DNA design is more easily detected than in a two T-DNA design. In the former case, the adjacent genome editing machinery is likely still present for TaqMan quantification and can inform progeny analysis. By contrast, for a T-DNA featuring solely donor template, only small left and right border fragments will remain following Cas12a cleavage which are more challenging to detect by standard PCR-based assays. This is further underscored by the likelihood that the error-prone NHEJ pathway repairs the DSBs following donor release at the T-DNA. As a result, it is possible that clean T0 events using two T-DNA designs may still retain some T-DNA border residues in the genome.

To address limitations of PCR-based assays for evaluating clean insertions, we further analyzed several events using deep sequencing to confirm that there is no presence of unwanted exogenous transgenic sequences using a “No Exogenous DNA Edited Line (NEDEL)” analysis process. The NEDEL analysis utilizes Illumina-NGS based DNA capture methods and subsequent computational analysis to determine the presence or absence of unwanted extraneous DNA sequences in the plant genome (see Methods section). All material analyzed comprised TaqMan assay-verified clean T1 or BC1 progeny of events generated from biolistics experiments. Events from this delivery method were chosen since it is more likely to generate undesirable additional insertions. The results of this analysis show that every event contained at least one T1 or BC1 plant with no exogenous DNA, and in most cases (16 out of 18 DNA biolistic events and five out of 6 RNP biolistic events), all analyzed plants were NEDEL-confirmed (Supplementary Table 10). It is important to note that the probe size used for NEDEL analysis is 120bp. A study using similar capture approaches (Zastrow-Hayes et al., 2015) reported that fragment sizes as small as 35 bp could be detected and 50 bp fragments reliably detected. Therefore, it is possible that smaller vector backbone fragments less than 50 bp could remain undetected by this assay. Alternative approaches such as whole-genome sequencing are recommended to capture information related to integration of smaller backbone fragments.

### Targeted insertion event characterization via nanopore sequencing reveals contributions by end-joining pathways

While this study largely seeks to enrich for precise HDR events generated with maize immature embryo explants, other end-joining pathways are known to operate throughout all phases of the cell cycle. Although we observed that TIN event generation wholly through NHEJ or MMEJ is not efficient for multi-kb sequences (Table 1), it is possible that many TIN-positive events might have been repaired by HDR on only one side, as reported previously for DNA double strand break repairs (Puchta, 1998). For example, recent work in Arabidopsis leveraging HDR-mediated targeted insertion used whole-genome sequencing to identify a variety of unexpected outcomes indicative of NHEJ repair, including tandem T-DNA and donor insertions (Mudgett et al., 2024).

**FIGURE 2:**
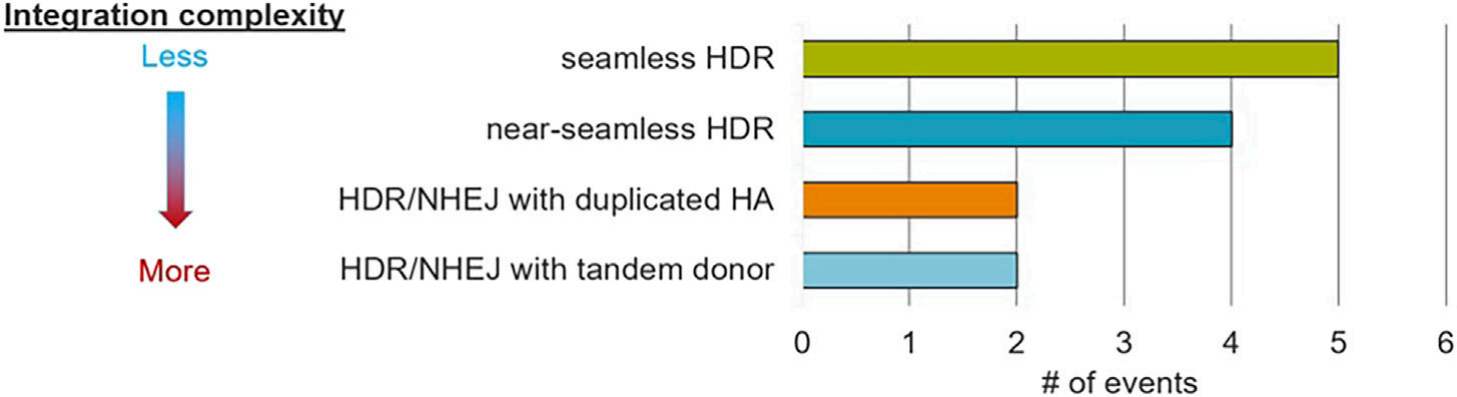
DNA repair outcomes for 13 TIN events from pSYN28315 and pSYN28303 analyzed by nanopore sequencing. In terms of integration complexity, outcomes range from seamless HDR (least complex) insertions to mixed HDR/NHEJ events containing tandemly integrated donor molecules (highly complex).

To more precisely understand insert intactness in our TIN events, we conducted overlapping PCR assays at the left and right junctions for each of the previously generated djTIN-positive pSYN28303 and pSYN28315 events (Table 3). A total of 13 such events yielded amplicons with consistently strong bands in both junction assays; these samples (comprising two replicate BC1 plants per event) were taken forward to nanopore sequencing. Several kinds of repair outcomes were identified across these events (Figure 2; Supplementary Table 11), including completely seamless repair on both sides (5 events), near-seamless integrations with one or more indels present in a homology arm or trait sequence (4 events), mixed HDR/NHEJ integrations featuring one duplicated homology arm (2 events), and tandem donor insertions (2 events). The last and most complex outcome appears to feature HDR integrations of two different donor molecules on each side of the target (the estimated PMI copy number in all such cases being equal to or greater than 2), with NHEJ repair likely responsible for ligating the two donors together. Of the 10 clean events from Table 4, all featured integration outcomes from the first three insertion categories, including no unwanted transgenic sequences, further confirming their status as clean events.

The observation of near-seamless integrations for four of the events is difficult to explain. Although HDR is much less error-prone than NHEJ, its dependence on *de novo* synthesis does not exclude errors generated by DNA polymerases. Alternatively, repair using MMEJ could potentially contribute to these errors since this mechanism frequently generates indels near the DSB site. Although the donor templates used in this study featured 500 bp homology arms, these molecules were nonetheless designed to be released by Cas12a cleavage and therefore are potentially subject to degradation at either end by endogenous exonucleases. In such a scenario, smaller arms more suitable for MMEJ repair might instead be generated.

The two tandem donor insertion events were further analyzed using nanopore whole-genome sequencing with adaptive sampling (Supplementary Figure 1; Supplementary Table 12, see Methods). Consensus reads spanning both junctions of event MZKE224602B079A identified three tandem copies of donor sequence and the presence of a single copy of pSYN28315 backbone. A consensus sequence for the second event MZKE231027A011A could not be assembled across both junctions, however data across individual junctions revealed at least five donor copies of varying sizes at the target site. Additional features including editing machinery T-DNA copies and small segments of *Agrobacterium* chromosomal DNA were also detected.

The observed copy number for event MZKE231027A011A contrasts with the two donor copies detected in the original T0 plant (Supplementary Table 8). Several ZmSH1gRNA2 residues were also detected, leading to the possibility that additional rounds of Cas12a cleavage and homology-directed repair occurred at this site. This scenario could lead to tandem duplication of multiple copies of donor sequence, a phenomenon observed in many gene families (Barco and Clay, 2019).

*Agrobacterium* chromosomal DNA insertions into plants have been previously described (Ülker et al., 2008); we could not determine whether these sequences were already rearranged in *Agrobacterium* cells before T-DNA transfer or were independently transferred and co-integrated. Altogether these results provide confirmation of complex and highly repetitive insertions and showcase the capabilities and limitations of nanopore adaptive sequencing technology.

Collectively these data suggest that relative to other pathways, HDR is largely active in maize immature embryos for directing large sequence insertions. This contrasts with results from other studies that evaluated combinations of different repair outcomes by similar means (Suzuki et al., 2019; Mudgett et al., 2024). However, in general the perfect HDR insertion rates described here remain low overall (<5%), in agreement with observations from many groups (Gao, 2021).

## Conclusion

Targeted large sequence insertion is a powerful tool in plant biotechnology, revolutionizing the ability to modify plant genomes with precision and enabling consistent gene expression and trait performance across events (Gao et al., 2020; Sun et al., 2024). In this report, we have outlined approaches to successfully enrich for targeted insertion events in maize. These include molecular designs featuring long homology arms and multiple T-DNAs, as well as a variety of gene delivery methods. To our surprise, we observe higher rates of large sequence integrations mediated by HDR than NHEJ or MMEJ pathways. Although we ultimately embrace *Agrobacterium*-mediated delivery as a workhorse method, the high efficiencies we observed with biolistics, coupled with its many advantages (e.g., broad host range, capacity for larger DNA inserts) show it remains an effective platform for targeted insertions. Additionally, we demonstrate the effectiveness of nanopore sequencing as a rapid low-cost NGS method capable of evaluating the intactness of large insertions in plants.

While many efforts including those outlined here rely heavily on native DNA repair mechanisms, alternative approaches such as CRISPR-based prime editing derivatives PASTE, Twin-PE, Primeroot, and PCE/RePCE (Anzalone et al., 2019; 2022; Yarnall et al., 2023; Sun et al., 2024; 2025) offer ways to largely bypass these limitations, potentially increasing efficiency. However such methods require additional machinery components, which may complicate their use in some plant delivery systems compared to CRISPR RNP alone. Moreover, RePCE technology’s enablement of seamless large sequence integrations requires at least three sequential editing steps at four targets (recombinase site integration, large cargo integration, and re-editing to remove scars at both junctions). The degree to which such seamless insertion technologies can perform compared to nuclease-mediated HDR remains to be seen.

Low efficiencies for HDR-based targeted insertion can be improved by including the use of morphogenic factors such as BABYBOOM or WUSCHEL (Peterson et al., 2021). Another study demonstrated that sequential transformation of DNA donor into parental lines expressing Cas9 from egg cell and early embryo-specific promoters can improve the HDR efficiencies in Arabidopsis (Miki et al., 2018). As insertion and other editing technologies continue to evolve, they open new avenues for crop improvement, sustainable agriculture, and food security, reshaping the future of plant breeding and genetic engineering.

## Materials and methods

### Etiolated maize leaf protoplast isolation, transient transfection, and transient editing analysis

Protoplast isolation, RNP-mediated transfection, and transient genome editing detection were carried out as previously described (Dong S. et al., 2021; Rigoulot et al., 2023). Primers used for editing detection by amplicon sequencing are listed in Supplementary Table 13.

### Plant materials

Syngenta’s proprietary elite maize inbred variety NP2222 was used in all experiments. NP2222 plants were grown in a greenhouse and immature embryos were isolated and used for biolistics- and *Agrobacterium*-mediated transformation as described previously (Wright et al., 2001; Zhong et al., 2018).

### gRNA selection and transient chromosomal target cleavage assay

Potential gRNAs with a TTTV PAM were identified in the target region and moderate GC content (30%–80%) was used as the first filter to eliminate gRNAs with very high AT or GC content. The remaining gRNAs were subjected to gRNA structure and potential off-target analyses (Bae et al., 2014; Jensen et al., 2017), only a subset of gRNAs with minimal stable secondary structure in the spacer region and off-target sites are selected for target cleavage assay with protoplast and RNP (Dong S. et al., 2021). Cas12a RNP complex preparation, etiolated maize leaf protoplast isolation and transfection and chromosomal target cleavage assays were carried out as described previously (Dong and Ronald, 2021). Cas12a (aka. Cpf1) enzymes including AsCas12a-Ultra and LbCas12a-Ultra (Supplementary Table 1) (Behlke et al., 2018; Vakulskas et al., 2020; Zhang et al., 2020) and gRNAs were purchased from IDT (Integrated DNA Technologies, Inc.). The gRNA scaffold used for LbCas12a is based on CRISPR-LbCpf1 system, while the gRNA scaffold used for AsCas12a is based on CRISPR-AsCpf1 system (Zetsche et al., 2015). The coding sequences of LbCas12a, Mb2Cas12a and their variants were optimized with maize codons and contain an optimized long linker 6x(GGGS) between the SV40 NLS at the N-terminus and a short linker with 2 NLS at the C-terminus (GSPKK KRKVS GGSSG GSPKK KRKV). eMb2Cas12a is an engineered Mb2Cas12a with long linker, bridge helix (BH) and nuclease domain swap and a potato ST-LS1 intron with improved editing activity (Geng et al., 2024).

### Vector design, construction and donor amplification

For vector construction, backbone fragments from base vectors were digested with restriction enzyme(s) (NEB and Thermo Fisher Scientific). Insert fragment(s) were amplified from existing vector templates by PCR using CloneAmp™ HiFi PCR Premix (Takara) or PrimeSTAR® GXL DNA Polymerase (Takara). In cases where fragments lacked existing vector templates, they were synthesized from IDT or Genscript. All fragments are assembled with NEBuilder® HiFi DNA Assembly Master Mix (NEB). Final vectors were screened with restriction enzymes and confirmed with NGS.

Donor DNA fragments used in biolistic experiments were PCR-amplified using primers containing 5′-phosphate and phosphorothioate modifications (IDT, Supplementary Table 14).

### Maize transformation and molecular analysis of targeted insertion events

Maize transformation for generation of stable transgenic events was performed as described (Zhong et al., 2018; Dong S. et al., 2021). For analysis of editing and transgenesis in regenerated plants, leaf samples were harvested, and total genomic DNA was extracted from rooted plants. TaqMan assays were used to detect events positive for target site mutations and transgenes (Ingham et al., 2001; Chen et al., 2018). To confirm integrations, all positive events for targeted insertion on one or both sides were resampled and analyzed via short gel-based junction-PCR assays. For the analysis of T+ generation of targeted insertion plants, TaqMan assays followed by gel-based junction-PCR assays were performed. To determine the full donor insertion, overlapping gel-based junction-PCR and whole donor gel-based junction-PCR were performed. 2x Platinum SuperFi II Green PCR Master Mix (Invitrogen) and PrimeSTAR^®^ GXL DNA Polymerase (Takara Bio Inc.) were used for PCR amplification by following manufacture protocol. PCR amplicons were purified by Ampure XP beads (Beckman Coulter Life Sciences) and sequenced by Oxford Nanopore sequencing (Plasmidsaurus Inc.) to fully characterize the type of targeted insertions.

### No exogenous DNA Edited Line (NEDEL) analysis

NGS library construction, DNA capture, and sequencing referred to industrial standard protocols (Gnirke et al., 2009; Mamanova et al., 2010; DuBose et al., 2013; NimbleGen SeqCap EZ Library LR User’s Guide [2011]; KAPA HyperCap Workflow v3.0 [Roche Sequencing Solutions, Inc. 2015-2020]; xGen hybridization capture of DNA libraries for NGS target enrichment [IDT, version 4 May 2019]). Biotinylated probe panel was custom designed with 3x tiling probes covering almost all the nucleotides of the constructs. Individual probes were synthesized and pooled together by IDT following their standard protocol. Genomic DNA was extracted and purified from maize leaf materials using Promega’s MagneSil Paramagnetic bead-based method. DNA quantification and qualification was performed with Fragment Analyzer (Advanced Analytical Inc.). Following Illumina’s library preparation protocol, DNA was enzymatically fragmented to peak size around 300–500 bp. Fragmented DNA was end repaired, A-Tailed, and ligated to standard Illumina adapters containing unique barcodes. PCR amplified DNA libraries were checked with SpectraMax (Molecular Devices) or TapeStation (Agilent Technologies), and pooled in equal molar ratios for target capture. The target enrichment was accomplished according to xGen hybridization capture of DNA libraries for NGS target enrichment (IDT, version 4 May 2019). The prepared fragment DNA library was mixed with universal blockers to prevent adapter to adapter hybridization. Blocked library fragments were then annealed to the 5′ biotinylated oligonucleotide probes at 65 °C for 4–16 h after a quick denaturation. Following the hybridization, streptavidin-coated magnetic beads were added to the mixture to bind to probes hybridized to target DNA. The captured DNA fragments were washed following the standard protocol, then eluted from the beads and amplified with a few cycles of PCR. Post-capture PCR amplicons were purified with magnetic beads and sequenced on Illumina NextSeq 1000 or other Illumina sequencing platform with paired end reads.

The generated sequence reads were used for NEDEL confirmation using a bioinformatics analysis pipeline developed by Syngenta. This pipeline aligns construct aligning reads to a reference comprised of transformation constructs and previously identified wild type genome sequences with similarity to construct sequences. It then uses these alignments (both partial and full) to ascertain whether any reads bearing high similarity to the transformation construct(s) are indeed derived from the integration of construct sequences (both wanted and unwanted) in the plant genome or not.

## Data Availability

The datasets used and/or analyzed in this article are available from the corresponding authors on reasonable request.
