# Supplementary material for: Development of efficient targeted insertion mediated by CRISPR-Cas12a and homology-directed repair in maize
Source: Front Genome Ed. 2025 Dec 4;7:1713347. doi: 10.3389/fgeed.2025.1713347 (PMC12711819; doi:10.3389/fgeed.2025.1713347)
Supplement: Supplementary file 1 [file Supplementaryfile1.docx]

**Supp Table 1**: Protoplast editing rates and leaf methylation signal for selected gRNAs at the ZmSH1 locus. Methylation data featured 15x or greater coverage.

| gRNA name | LbCas12a Ultra, amp-seq  Editing rate% | gRNA target sequence | Methylation signal % | | |
| --- | --- | --- | --- | --- | --- |
|  |  |  | CpG | CHG | CHH |
| Bx9TS2 | 58.3 | GCACCAAGGAAGGGGAGGAGATC | 0.0 | 0.0 | 0.0 |
| ZmSH1gRNA2 | 49.6 | GCATGTGAAGGAACCCGAACCAG | 0.0 | 2.7 | 0.4 |
| ZmSH1gRNA6 | 63.0 | GCGGCCGGCCCTTATCTCCTTCC | 0.5 | 0.0 | 0.1 |
| ZmSH1gRNA15 | 0.0 | GCTATTTCTTGGGTTTTGTTTGT | 99.1 | 86.8 | 1.9 |
| ZmSH1gRNA16 | 37.8 | TAACATAGTTCATCCTCCCATTT | 96.3 | 90.7 | 2.5 |
| ZmSH1gRNA17 | 0.0 | TTGGGTTTTGTTTGTAGGCCTTG | 99.1 | 86.8 | 1.9 |
| ZmSH1gRNA18 | 0.0 | TTTGTAGGCCTTGTCGTTATGCT | 99.1 | 86.8 | 1.9 |
| ZmSH1gRNA19 | 0.0 | TAGGCCTTGTCGTTATGCTCACA | 99.1 | 86.8 | 1.9 |
| ZmSH1gRNA20 | 0.0 | TTCAACTTAGAAACCGAAACATC | 97.7 | 87.6 | 2.2 |
| ZmSH1gRNA21 | 41.8 | GTGCATGCGCCCTTCCTGATGAA | 96.2 | 88.5 | 2.4 |
| ZmSH1gRNA22 | 35.3 | TTGCCCATGCTTCCCCGGCTCAG | 98.1 | 72.9 | 5.0 |
| ZmSH1gRNA23 | 25.3 | TCCGCCTCTCGAGAGCCGAACTT | 98.1 | 72.9 | 5.0 |
| ZmSH1gRNA24 | 48.4 | TTTATAGACCTTCAAGTTTGTCC | 98.1 | 72.9 | 5.0 |
| ZmSH1gRNA25 | 11.4 | TTTATCGCGGGTATCGCCAGCCA | 2.3 | 2.9 | 0.4 |
| ZmSH1gRNA26 | 43.0 | ATGGGCTGGTTGAAGGCCCGGGC | 0.2 | 0.0 | 0.5 |
| ZmSH1gRNA27 | 73.0 | GGAGCCAAACGCCAAACCCGCTC | 0.2 | 0.0 | 0.5 |
| ZmSH1gRNA28 | 51.7 | GCGTTTGGCTCCCAAAACCCGAT | 0.2 | 0.0 | 0.5 |
| ZmSH1gRNA29 | 54.8 | GCTCCCAAAACCCGATGGGCGCG | 0.2 | 0.0 | 0.5 |
| ZmSH1gRNA30 | 71.9 | GTAGTGGAAGGCACCGCGCCCAT | 0.1 | 0.3 | 0.3 |
| ZmSH1gRNA32 | 38.4 | AGGTTTGGGTGCCAATATTTTTT | 0.0 | 0.7 | 0.0 |
| ZmSH1gRNA33 | 53.8 | CCCCTTCCTCCACTCACGCTCGA | 0.9 | 4.4 | 0.5 |
| ZmSH1gRNA34 | 46.9 | TCTTTGCCGACGACCGCCATTTG | 0.9 | 4.4 | 0.5 |
| ZmSH1gRNA35 | 41.1 | CCGACGACCGCCATTTGTGCTCC | 0.9 | 4.4 | 0.5 |
| ZmSH1gRNA36 | 22.0 | TGCTCCTCTTCTCTTCCATCTCG | 0.5 | 2.2 | 0.3 |
| ZmSH1gRNA37 | 23.7 | TTGTCGAGCCCGTAGGTGATGGC | 0.0 | 2.3 | 1.2 |

Note: Methylation data featured 15x or greater coverage.

**Supp Table 2.** T0 stable genome editing efficiencies at selected maize chromosomal targets.

| **Construct** | **gRNA** | **Explants** | **No. of PMI+ events (TF%)** | **No. of edited events (Editing rate%)** |
| --- | --- | --- | --- | --- |
| pDEO26117 | ZmSH1gRNA2 | 1758 | 148 (8.4%) | 124 (83.8%) |
| pDEO26110 | ZmSH1gRNA6 | 1875 | 167 (8.9%) | 96 (57.5%) |
| pDEO26757 | ZmSH1gRNA18 | 1985 | 60 (3.0%) | 15 (25.0%) |
| pDEO26758 | ZmSH1gRNA32 | 2561 | 138 (5.4%) | 92 (66.7%) |

* Data from a single round of transformation

**Supp Table 3.** Targeted insertion (TIN) efficiency at maize chromosomal target ZmSH1gRNA2 using biolistics DNA delivery.

| **HOM arm length (bp)** | **Insert size (bp)** | **Construct** | **Experiment** | **Explants** | **PMI+ events (TF%)** | **Edited events (rate %)** | **Any junction PCR+ TIN events (ajTIN rate%)** | **Double junction PCR+ TIN events (djTIN rate%)** |
| --- | --- | --- | --- | --- | --- | --- | --- | --- |
| 526 / 507 | 7508 | pSYN25576; pSYN25633 | 5 | 5665 | 775 (13.7%) | 323 (41.7%) | 48 (6.2%) | 11 (1.4%) |
| 55 / 55 | 7508 | pSYN25576; pSYN25583 | 4 | 4679 | 431 (9.2%) | 110 (25.5%) | 15 (3.5%) | 0 (0%) |

Note: Transformation frequency (TF%) = (Number of PMI-positive events /Number of starting explants) x 100%; Editing rate ( %) = (Number of edited events / Number of PMI-positive transformants) x 100%; Any-junction targeted insertion rate (ajTIN%) = (Number of PCR positive events at either left or right junction / Number of any copy events) x 100%; Double-junction targeted insertion rate (djTIN%) = (Number of both junction PCR positive events/ Number of any copy events) x 100%.

**Supp Table 4** TIN efficiency at maize chromosomal target ZmSH1gRNA2 using biolistic co-delivery of LbCas12a RNP and a donor DNA restriction fragment.

|  | **Insert size (bp)** | **Construct** | **Experiments** | **Explants** | **PMI+ events (TF%)** | **Double junction PCR+ TIN events (djTIN%)** |
| --- | --- | --- | --- | --- | --- | --- |
|  | 6,177 | pSYN26389 | 2 | 3996 | 1063 (26.6%) | 10 (0.9%) |
|  | 7,508 | pSYN25633 | 11 | 12045 | 1433 (11.9%) | 8 (0.6%) |
|  | 10,182 | pSYN26413 | 4 | 8633 | 585 (6.8%) | 2 (0.3%) |

Note: For all constructs, the left and right homology arm lengths were 526nt and 508nt respectively Transformation frequency (TF%) = (Number of PMI-positive events /Number of starting explants) x 100%; Double junction targeted insertion rate (djTIN%) = (Number of both junction PCR positive events/ Number of any copy events) x 100%.

**Supp Table 5** Effect of donor template amount on targeted insertion rates using biolistic co-delivery of LbCas12a RNP and a donor DNA restriction fragment.

| Donor DNA/plate | Experiments | Explants | PMI+ events (TF%) | Edited events (rate%) | Double junction PCR+ TIN events (djTIN rate%) |  |  |  |
| --- | --- | --- | --- | --- | --- | --- | --- | --- |
| 0.38 pmol | 2 | 1993 | 350 (17.6%) | 255 (72.9%) | 5 (1.4%) |  |  |  |
| 0.76 pmol | 2 | 1571 | 264 (16.8%) | 219 (83.0%) | 12 (4.6%) |  |  |  |

Note: Transformation frequency (TF%) = (Number of PMI-positive events /Number of starting explants) x 100%; Editing rate (%) = (Number of edited events / Number of PMI-positive transformants) x 100%; Double junction targeted insertion rate (djTIN%) = (Number of PCR positive events at both left and right junctions / Number of edited transformants) x 100%.

**Supp Table 6.** Effect of donor end modifications on targeted insertion rates using biolistic co-delivery of LbCas12a RNP and a donor DNA restriction fragment.

| Treatment | Donor Construct | Experiments | Explants | PMI+ events (TF%) | Edited events (rate%) | Double junction PCR+ TIN events (djTIN rate%) |  |  |  |
| --- | --- | --- | --- | --- | --- | --- | --- | --- | --- |
| Donor with unmodified backbone | 26130 | 3 | 3182 | 1047 (32.9%) | 410 (39.2%) | 2  (0.2%) |  |  |  |
| Donor with phosphothioate- modified backbone | 26130 | 3 | 2896 | 945 (32.6%) | 314 (33.2%) | 9  (1.0%) |  |  |  |

Note: Transformation frequency (TF%) = (Number of PMI-positive events /Number of starting explants) x 100%; Editing rate (%) = (Number of edited events / Number of PMI-positive transformants) x 100%; Double-junction targeted insertion rate (djTIN%) = (Number of double junction PCR positive events/ Number of any copy events) x 100%.

**Supp Table 7** Evaluation of Mb2Cas12a editing rates per PMI+ and Cas12a+ event for the constructs in Table 3.

| **Construct** | **Cas12a ortholog and mutant version** | **Design** | **PMI+ and Cas12a+ events** | **PMI+ and Cas12a+ events with edits (rate%)** |
| --- | --- | --- | --- | --- |
| pSYN27848*^,^** | Mb2Cas12a WT | 1T2C | 142** | 115 (81.0%) |
| pSYN28303 | Mb2Cas12a “Opt1” | 1T2C | 294 | 252 (85.7%) |
| pSYN28304 | Mb2Cas12a “Opt2” | 1T2C | 577 | 465 (80.6%) |
| pSYN28315 | Mb2Cas12a “Opt1” | 2T2C | 307 | 264 (86.0%) |
| pSYN28316 | Mb2Cas12a “Opt2” | 2T2C | 296 | 237 (80.1%) |
| pSYN27413* | LbCas12a D156R | 2T2C | 76 | 62 (81.6%) |

* The potato ST-LS1 intron is present in the Cas12a CDS
**Two experiments did not capture the Cas12a insertion (Cas12a+) data

**Supp Table 8** Molecular analysis summary of T0 and BC1 plants derived from putative TIN events of pSYN28315 and pSYN28303 constructs.

Note: T0 (both arm) = double junction positive by taqman assay; sJPCR (both arm) = double junction positive by short-junction PCR assay; T0 (PMI) copy number = detected by taqman assay; JPCR: OL-L and OL-R: “Y” or “yes” denotes positive at the left or right junction by overlapping gel-based junction PCR. “N” denotes the absence of a band. The designations “larger,” “weak,” or “multiple bands” denote additional characteristics for a positive band relative to the expected amplification assuming HDR repair. “nt” denotes not tested due to negative results achieved at both sides in the junction taqman assay.

**Supp Table 9** Chi-square analysis of TaqMan-analyzed targeted insertions from BC1 progeny derived from the 10 clean TIN events in Table 4.

| **Event** | **Construct** | **Total plants analyzed** | **Observed plants, positive calls** | **Observed plants, negative calls** | **chi-square value** | **p-value** |
| --- | --- | --- | --- | --- | --- | --- |
| MZKE224604A110A | pSYN28315 | 25 | 15 | 10 | 1.00 | 0.32 |
| MZKE224803A003A | pSYN28315 | 27 | 12 | 15 | 0.33 | 0.56 |
| MZKE224604A031A | pSYN28315 | 32 | 14 | 18 | 0.50 | 0.48 |
| MZKE224602B007A | pSYN28315 | 30 | 15 | 15 | 0 | 1.00 |
| MZKE224604A165A | pSYN28315 | 32 | 18 | 14 | 0.50 | 0.48 |
| MZKE224604A021A | pSYN28315 | 31 | 17 | 14 | 0.29 | 0.59 |
| MZKE231027A002A | pSYN28315 | 32 | 12 | 20 | 2.00 | 0.16 |
| MZKE231221A051A | pSYN28315 | 28 | 15 | 13 | 0.14 | 0.71 |
| MZKE224603A158A | pSYN28303 | 32 | 17 | 15 | 0.13 | 0.72 |
| MZKE224603A134A | pSYN28303 | 21 | 11 | 10 | 0.048 | 0.83 |

**Supp Table 10** NEDEL analysis of TaqMan assay-verified clean T1 or BC1 plants.

| **T0 TIN event ID** | **Donor** | **Editing machinery construct** | **Number of BC1 plants analyzed** | **Number of clean TIN plants** | **Biolsitics delivery method** |
| --- | --- | --- | --- | --- | --- |
| MZKE212406A052A | pSYN26130 | pDEO26111 | 2 | 1 | DNA |
| MZKE211100A296A | pSYN26130 | pDEO26111 | 2 | 2 | DNA |
| MZKE211100A027A | pSYN26130 | pDEO26111 | 3 | 3 | DNA |
| MZKE211100A277A | pSYN26130 | pDEO26111 | 2 | 2 | DNA |
| MZKE211100A306A | pSYN26130 | pDEO26111 | 7 | 7 | DNA |
| MZKE211100A330A | pSYN26130 | pDEO26111 | 3 | 3 | DNA |
| MZKE211100A423A | pSYN26130 | pDEO26111 | 8 | 6 | DNA |
| MZKE211207A134A | pSYN26130 | pDEO26111 | 3 | 3 | DNA |
| MZKE212406A052A | pSYN26130 | pDEO26111 | 2 | 2 | DNA |
| MZKE212803A173A | pSYN26130 | pDEO26111 | 2 | 2 | DNA |
| MZKE212601A091A | pSYN26130 | pDEO26111 | 2 | 2 | DNA |
| MZKE212803A186A | pSYN26130 | pDEO26111 | 1 | 1 | DNA |
| MZKE212601A112A | pSYN26130 | pDEO26111 | 1 | 1 | DNA |
| MZKE212702A071A | pSYN26130 | pDEO26111 | 3 | 3 | DNA |
| MZKE212702A076A | pSYN26130 | pDEO26111 | 4 | 4 | DNA |
| MZKE212702A127A | pSYN26130 | pDEO26111 | 1 | 1 | DNA |
| MZKE212704A090A | pSYN26130 | pDEO26111 | 2 | 2 | DNA |
| MZKE212802A040A | pSYN26130 | pDEO26111 | 2 | 2 | DNA |
| MZKE211600A324A | pSYN26389 | NA | 2 | 2 | RNP |
| MZKE212001B146A | pSYN26389 | NA | 2 | 2 | RNP |
| MZKE211700B019A | pSYN26413 | NA | 2 | 2 | RNP |
| MZKE211803A093A | pSYN26413 | NA | 2 | 2 | RNP |
| MZKE210309B214A | pSYN26130 | NA | 2 | 1 | RNP |

Note: In the case of T0 event MZKE211100A027A, T1 plants were generated via selfing. For all other lines, BC1 plants were generated from backcrossing to WT maize plants.

**Supp Table 11** TaqMan, PCR, and nanopore sequencing analysis summary of BC1 plants from selected pSYN28315 and pSYN28303 TIN events.

Note: T0 (both arm) = double junction positive by taqman assay; sJPCR (both arm) = double junction positive by short-junction PCR assay; T0 (PMI) copy number = detected by taqman assay; JPCR OL-L and OL-R: “Y” or “yes” denotes positive at the left or right junction by overlapping gel-based junction PCR. “N” denotes the absence of a band. LHA/RHA denote the left/right homology arm. prCMP denotes the promoter driving the GOI. The designations “larger” and/or “weak” denote additional characteristics for a positive band relative to the expected amplification assuming HDR repair. NGS OL-L and OL-R: SDN1 denotes a likely mutation of the gRNA sequence by the Cas12a enzyme.

**Supp Table 12** Nanopore adaptive sampling summary for the analysis of two tandem donor TIN events.

**Suppl Table 13** Maize target sequences and primers for transient editing analysis by amplicon sequencing and for donor DNA amplification.

| **gRNA name** | **forward primer name** | **forward primer sequence** | **reverse primer name** | **reverse primer sequence** |
| --- | --- | --- | --- | --- |
| All ZmSH1 guides | LZ222 | CGAGGAGAAGCAGTTCTTGA | LZ223 | AACATCCTTGCAGACGGTAT |
| Bx9TS2 | LZ196 | AAACACTAAACACTCCCCTCTG | LZ197 | GTTTACCCATCTCTTTTAACACTAT |

**Supp Table 14.** Primers used for donor DNA amplification. Asterisks denote phosphorothioate linkages.

| **name** | **sequence** | **template vector** |
| --- | --- | --- |
| CJ104F | G*A*TCCGTTTCCGTGCATGATTAG | pSYN26130 |
| BB080 | T*G*TGGCTAATAACAGTGGTCA | pSYN26130 |
| CJZ9R | C*T*TCACTAACCACTAGCGCTTG | pSYN25633, pSYN26413, pSYN26389, pSYN27413 |
| CJZ15R | C*G*CTCCTGTGGTGGAGATC | pSYN25633, pSYN26413, pSYN26389, pSYN27413 |

**Supp Figure 1.** Schematic of all-in-one Cas12a editing machinery and donor constructs for Agrobacterium-mediated maize transformation featuring 1-T-DNA (top) or 2-T-DNAs (bottom) to deliver Cas12a editing machinery cassettes and donor, and using 2 Cas12a-mediated cuts to release donor sequences. HA, homology arm of varying size. Scissors indicate ZmSH1gRNA2 cut sites to enable donor release. prSoUbi4, sugarcane Ubiquitin-4 promoter; Cas12a, maize codon-optimized eMb2Cas12a coding sequence; tNOS, NOS terminator; prOsU6, rice U6 promoter; prAct1, rice actin-1 promoter; GOI, gene of interest; PMI, phosphomannose isomerase gene sequence; RB, T-DNA right border; LB, T-DNA left border.


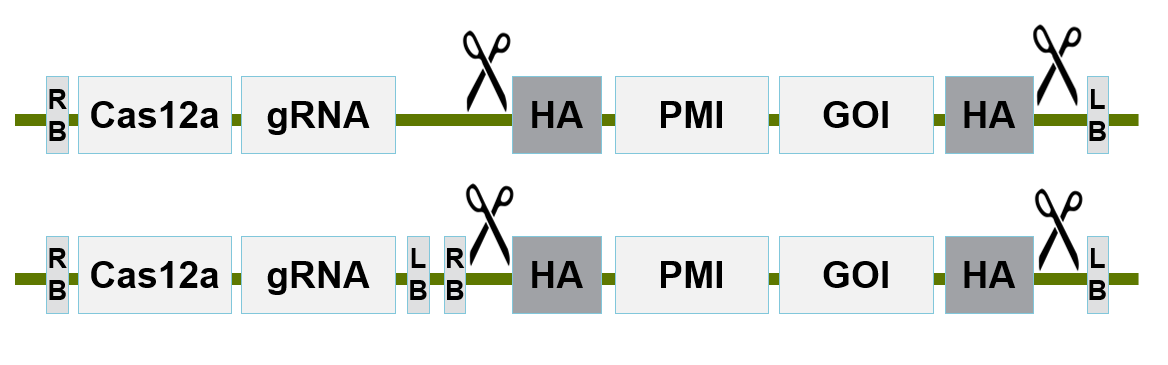


**Supp Figure 2.** Complex inserts in the two tandem donor TIN events based on nanopore adaptive sequencing analysis. Grey, blue, red, and yellow annotations respectively denote NP2222 target site-adjacent genomic DNA, full/partial donor sequences, other pSYN28315 vector sequences, and Agrobacterium genomic DNA.

**
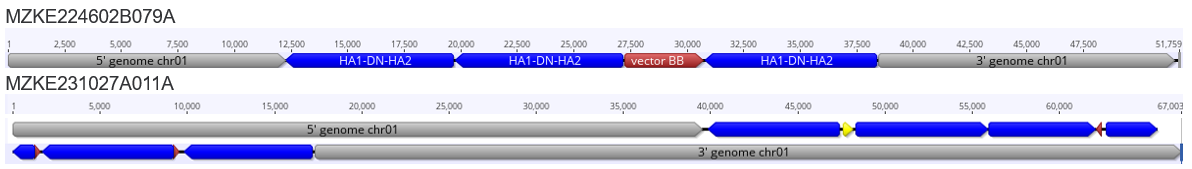
**
